# An Introduction to Structural Competency for Haitian-Identified Patients: History, Culture, and Access to Care

**DOI:** 10.15766/mep_2374-8265.11207

**Published:** 2021-12-27

**Authors:** Christina Pardo, Nicholas Brutus, Dorah Labatte, Florence Saint-Jean, Sophia Tribié, Ivrose Joseph, Camille A. Clare, Julia Su, John Paul Sánchez

**Affiliations:** 1 Assistant Professor, Department of Obstetrics and Gynecology, SUNY Downstate Health Sciences University; 2 First-Year Medical Student, Albany Medical College; 3 Research Specialist, SUNY Downstate Health Sciences University; 4 Executive Director, Global Trauma Research; 5 Second-Year Resident, Department of Family Medicine, SUNY Downstate Health Sciences University; 6 Fourth-Year Resident, Department of Obstetrics and Gynecology, SUNY Downstate Health Sciences University; 7 Associate Professor, Department of Obstetrics and Gynecology, and Associate Dean of Diversity and Inclusion, New York Medical College; 8 Third-Year MD/PhD Candidate, Donald and Barbara Zucker School of Medicine at Hofstra/Northwell; National President, Latino Medical Student Association; 9 President, Building the Next Generation of Academic Physicians

**Keywords:** Social Determinants of Health, Community-Based Medicine, Cultural Competence, Diversity and Inclusion, Health Equity

## Abstract

**Introduction:**

The Haitian population within the US represents the largest diaspora outside of Haiti, with most Haitians residing in major urban communities. Despite clear differences in health outcomes specific to Haitians, the community has traditionally been aggregated into the general Black population. To address specific health disparities, this workshop was designed to distinguish and elaborate on the health care problems affecting Haitians.

**Methods:**

We created an interactive 60-minute workshop including a PowerPoint presentation, two case presentations, and a 5-minute informational video to bring awareness of the historical perspectives impacting Haitian/Haitian American health, access to care, and health care disparities to providers. Knowledge was assessed by pre- and postworkshop evaluation forms. The module was aimed at health care professional learners.

**Results:**

Seventy-four people with diverse ethnoracial identities, including medical students, residents, academic faculty, physicians, nonmedical graduate students, and health care staff and administrators, attended three workshops. All learning objectives were met, with pre- and postworkshop data indicating a statistically significant increase in participants’ reported confidence. Workshop attendees commented positively on the group discussion component, the workshop's interactive nature, the opportunity to apply taught knowledge to case presentations, and the historical context provided.

**Discussion:**

As the number of Haitian immigrants continues to rise throughout US urban communities, providers must increase their culture competency in training and delivery to improve care for a major population. This module can help better prepare health care providers and trainees to offer competent care to Haitian/Haitian American patients.

## Educational Objectives

By the end of this session participants will be able to:

1. Describe the history of Haiti and Haitians within the United States.
2. List the health issues and disparities of Haitians residing in Haiti and in the United States.
3. Explain how two implemented policies have affected Haitians’ and Haitian Americans’ health outcomes.
4. Describe two health care access problems faced by Haitians in the United States and/or Haiti.

## Introduction

The Haitian population represents the second-largest Black foreign-born population in the United States.^1^ Additionally, the Haitian population in the United States represents the largest outside of Haiti.^2^ The United States and Haiti have had a long and complex history of relations, which has led to a reciprocally significant presence in both countries.^2^ As it relates to health in the United States, the Haitian community is most often epidemiologically grouped within Black or immigrant populations for research purposes.^3,4^ This grouping has limited the relative number of publications dedicated specifically to Haitian health disparities. Much of the literature on the Haitian population has been focused on the health conditions prevalent in Haiti, such as HIV/AIDS, and, most recently, the impact of the 2010 earthquake.^5,6^ Studies on these health conditions within the Haitian community have demonstrated disparities in health outcomes compared to the Black population.^5,7^ Studies on cervical cancer, for example, have shown that Haitian immigrants are less likely to receive cervical cancer screening, are more likely to be diagnosed with cervical cancer at a higher stage, and have higher mortality rates compared to Black Americans.^7–9^

Racial and ethnic disparities are long-standing and have been recorded since population health statistics began being reported. As an institution, public health has recognized that these disparities are not a result of biology or individual factors, but rather of the social determinants of health and the structural inequities that cause them.^10^ While public health and health care institutions have navigated pathways towards mitigating the social and structural determinants of health to improve health outcomes, health care professionals have historically focused on cultural competency to care for diverse populations.^11^ Cultural competency aims to provide clinicians with the skill to manage cultural differences and knowledge of how culture can influence health. Cultural humility, which we believe to be a superior practice when working with diverse communities, prioritizes a lifelong commitment to self-evaluation and focuses on the patient-provider power imbalance and community partnership.^12^ There has been growing focus on integrating the social and structural determinants of health into health care education. Structural competency has been suggested as a framework to best prepare students and those in training on the social and structural determinants of health.^12^ Structural competency is defined as “the capacity for health professionals to recognize and respond to health and illness as the downstream effects of broad social, political and economic structures.”^13,14^

The social and structural determinants of health that cause racial and ethnic health disparities are historically rooted but continue to be perpetuated through persistent inequities. Haitian immigrants are most likely to reside in urban communities that have been historically disadvantaged and are themselves subject to the same barriers. In New York City, New York, for example, the single largest Haitian immigrant population is in Brooklyn, where community assessments have estimated that 23% of deaths could have been averted based on the economic profile of the district.^15^ The addition of immigrant status adds another layer of barriers, such as those related to language, insurance access, and legal status.^16^ In addition, the Haitian immigrant population has a unique history and culture that influence not only everyday lives but the perception of health as well. Lastly, the Haitian community is one that has been subject to stigma and bias that persist today.

The three largest Haitian populations within the United States are in Miami, Florida; New York City, New York; and Boston, Massachusetts.^17,18^ These cities are also home to a significant number of academic medical institutions. New York City, for example, has eight medical school campuses and is home to the second-highest Haitian population in the United States. Academic medical centers and public health groups have also had a presence in Haiti dating back decades. Haiti, which has been described as the “nongovernmental organization (NGO) nation,” has a significant number of American medical and public health professionals who have worked in Haiti and continue to do so.^19^ Despite the long history of US-Haiti relations and the significant US presence in Haiti, to date there has not been a medical curriculum dedicated to Haitian health and the barriers that contribute to Haitian health disparities.

*MedEdPORTAL* is an excellent resource for educators to find curricula on how to best integrate racial disparities and social determinants of health into medical education.^13^ As we work to navigate this path within our institutions, we must appreciate that marginalized communities are not monolithic. There remains an importance in understanding the uniqueness of the communities we serve. These distinctions are important in understanding the unique barriers of a given community and enabling the development and implementation of effective interventions. Such community enclaves have a unique history and culture that contribute to their determinants of health. As previously developed curricula geared towards Indigenous and Puerto Rican communities have demonstrated, centering on and understanding the values of a given community are important.^20,21^ The coauthors of this module felt that it was important to highlight the disparities present within the Haitian community using similar methodology. While the methodology that traditional cultural competency utilizes has limitations, there remains value in understanding the historical and cultural aspects of a given community in addition to its barriers to care. Culturally responsive care is essential in achieving health equity. A systematic review of the impact of cultural competence training has demonstrated not only improved knowledge, attitudes, and skills of health professionals but also improved patient satisfaction.^22,23^

This workshop teaches practitioners how to utilize the Kern six-step curriculum model for structure, implementation, and assessment.^24^ In the first step, our problem identification and general needs assessment entailed a review of current literature and speaking with community leaders on Haitian health care. For step 2, we reviewed prior *MedEdPORTAL* publications to understand how culture-specific content has been included in medical school curricula. Medical students and faculty were engaged from the beginning to provide constant direction for the development and implementation of this module. In step 3, we established our objectives and goals in accordance with the collaboration of our coauthors and the literature review. In step 4, our educational strategies consisted of an interactive PowerPoint presentation, case presentations, and a video highlighting the trauma experienced by Haitians. Each component included a reflection, problem-solving, understanding, and sharing of perspectives in an interactive group environment. In step 5, workshop implementation was presented during Black History Month (February 2020) for medical students, residents, physicians, faculty, and health care staff members at three different medical schools in urban and suburban New York. In step 6, evaluation and feedback were captured through pre- and postworkshop surveys completed by participants.

The aim of this workshop is to prepare participants to better care for the Haitian community. The unique history of Haiti and its relations with the United States is highlighted to show how that history contributes to federal policies, as well as stigma and bias that persist today. One does not need to be of Haitian background to facilitate this workshop. The module is designed for medical students, residents, institutional faculty, and physicians but is also appropriate for health care professionals, especially those who serve a Haitian population or travel to Haiti for work. This workshop offers an interactive curriculum presented through an equity lens and from the viewpoint of the Haitian community.

## Methods

This in-person workshop was written and modified by experts specializing in Haitian cultural research, health care disparities, and/or academic medicine. The primary team comprised six Haitian-identified individuals, all of them having experience working with Haitian communities in the United States and/or Haiti. The team consisted of two obstetrics and gynecology physicians, a family medicine physician, two graduate students, and an executive director of a global mental health organization. Christina Pardo facilitated and implemented the workshop at three medical schools in the state of New York (New York Medical College, Zucker School of Medicine at Hofstra/Northwell, and SUNY Downstate Health Sciences University). This workshop was offered at each of the above institutions as a part of their programming for Black History Month in February 2020. This educational study, which included students and residents, received institutional review board (IRB) approval at the sites of implementation (Rutgers Newark IRB, Pro20150001934-Academic Medicine Study). Data from the IRB-approved sites were interpreted using descriptive statistics paired to learner *t* tests to compare pre- and postassessment answers and a qualitative analysis based on the module's learning objectives to analyze open-ended questions.

This 60-minute workshop was created to increase the understanding of Haitian health care disparities and to fill a knowledge gap regarding cultural competency with Haitian and Haitian American patients. The targeted audience for this workshop included medical students, residents, and physicians but could also include other health care professionals and trainees. A fellow, faculty member, or administrator with robust experience in the social and structural determinants of health and/or a specific understanding of Haitian/Haitian American health issues would be the recommended facilitator. A facilitator without this experience should not be dissuaded from presenting this workshop. Rather, appropriate preparation and self-guided learning could prepare the facilitator to effectively present the material. Three primary educational approaches were featured: (1) a collaborative didactic PowerPoint presentation, (2) a video discussing trauma-informed care regarding Haitian patients, and (3) case-based discussion in small- and large-group formats highlighting common health problems and associated risk factors for Haitian patients.

The facilitator began with a didactic presentation (Appendix A) laying out the goals and learning objectives for the workshop. Using the speaker notes in the facilitator guide (Appendix B), the speaker walked through the PowerPoint (Appendix A) beginning with a geographic map, land acknowledgment, and a time line outlining Haitian history. The presentation then showed slides focused on Haitian culture and the relations between Haiti and the United States, including Haitian policy, causes for immigration, and challenges. The following segment of the presentation spoke about the concept of Haitian health, health issues faced in Haiti, and health issues faced by Haitians residing in the United States. Before showing the video (Appendix C), implemented in the workshop on slide 37 of the PowerPoint, the facilitator primed participants to consider how trauma could impact Haitian health. The facilitator then showed the video (Appendix C), which provided a breakdown of trauma to apply to lived experiences. The breakdown included information on the levels of barriers of care to reinforce concepts such as race inequity, cultural unfamiliarity, and negative Haitian stigma/bias. Participants were encouraged to reflect on and apply what had been presented in the video and throughout the PowerPoint to the case presentations (Appendix D). The facilitators worked through the case presentations on slides 40 and 41, applying the information already presented. A pre- and postworkshop survey was distributed to participants and used for revision and assessment of the developed workshop (Appendix E).

The following is an extensive description of the resources utilized to conduct the workshop successfully.

### Appendix A: Presentation

An overview of the workshop was outlined in a 45-slide PowerPoint. The presentation highlighted the core foundational concepts of the workshop, including key information about the social, historical, and structural determinants of health; a comprehensive analysis of Haitian health care disparities and health outcomes; and an overview of Haitian access to health care and policies/interventions affecting Haitians and Haitian Americans. Embedded within the PowerPoint presentation were the cases and a link to the Trauma in the Haitian Context video.

### Appendix B: Facilitator Guide

This document presented instructions on how to conduct the presentation and explanations of how to talk about each PowerPoint slide in detail. Facilitators who do not identify as Haitian/Haitian American and/or lack knowledge about Haitian culture, history, and/or values are recommended to follow the guide strictly and use the provided resources to better explain important concepts.

### Appendix C: Video Trauma in the Haitian Context

The video highlighted different levels of trauma experienced by Haitian or Haitian American patients. It is included here as Appendix C to reduce the possibility of encountering difficulties in downloading it from the internet during the presentation. The video was shown at slide 37.

### Appendix D: Case Presentations

These cases were developed to further elaborate on and discuss common health issues of and barriers to accessing quality care faced by Haitian and Haitian American patients. The case presentations were displayed on slides 40 and 41.

### Appendix E: Pre- and Postworkshop Evaluation Form

Participants were asked to fill out a pre- and postworkshop questionnaire. The primary questions collected demographic information and assessed participants’ competency levels regarding Haitian culture, Haitian/Haitian American history, and health care disparities affecting Haitian patients aligned with the workshop objectives.

Demographic questions were included on the preworkshop survey. Two open-ended short answer questions were asked on the postworkshop assessment: “What did you like about this workshop?” and “What suggestions do you have to improve this workshop?” Each participant was given approximately 5 minutes to complete the pre- and postworkshop evaluations.

The review of the PowerPoint, facilitator guide, video of trauma in the Haitian context, and case presentations took approximately 3 hours. We recommend that no more than two cofacilitators lead the workshop. Any additional facilitators should spend extra time in advance discussing the breakdown of slides between presenters by phone or face to face.

Materials needed included writing utensils, audiovisual equipment to display the PowerPoint presentation, computer with video and audio, printed copies of the case presentations, and printed copies of the pre- and postworkshop assessment forms.

## Results

The number of participants per session ranged from nine to 47. Seventy-four (100%) participants responded to the preassessment questionnaire, and 64 (86%) completed the postassessment. The three workshops’ respondents (*n* = 74) were a diverse sample by gender and by race and ethnicity: 36 (49%) identified as female, 29 (39%) as White, seven (10%) as Hispanic/Latino, 20 (27%) as African American/Black, and 14 (19%) as Asian. There were 39 medical students, 18 academic faculty members/staff, seven medical residents, and two nonmedical graduate students.

Respondents’ reported confidence in their ability to achieve each of the four stated learning objectives was greater after the workshop. Confidence was rated on a 0–4 scale (0 = *no confidence,* 4 = *complete confidence*). For all four objectives, the preworkshop median was 1, and the postworkshop median was 3. Through use of the Mann-Whitney *U* test, it can be concluded that the respondents’ confidence for each objective was statistically significantly higher after the workshop than before (*p* < .001).

Regarding the workshop's effect on the participants’ knowledge base, when comparing pre- and postworkshop questionnaires, a statistically significant increase in the correct answer being chosen (Table) was seen. An independent-samples *t* test showed a statistically significant difference of at least *p* < .05 for each question.

**Table.**
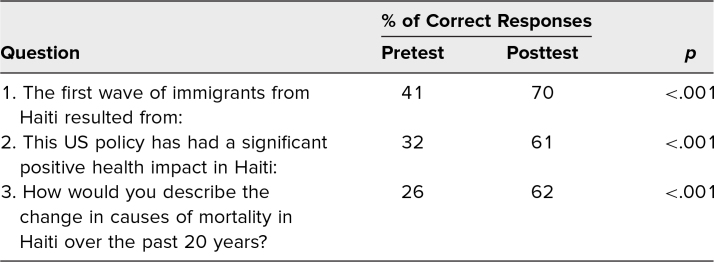
Percentage of Correct Responses per Question Pre- and Postworkshop (*N* = 74)

As illustrated in the comments below, participants reported that the workshop heightened their ability to describe Haitian history and Haitian identity, identify disparities of Haitians residing in the United States, explain policies impacting health outcomes of Haitians, and describe at least two health care access problems faced by Haitians and Haitians in the United States. An emerging theme was that participants enjoyed the workshop because of the use of media (e.g., videos, images, and historical time line), relevant historical context, and interactive cases that incorporated learning objectives into practical patient examples.

We arranged participants’ responses by each learning objective for the questions “What did you like about this workshop?” and “What suggestions do you have to improve this workshop?” Remarks on the workshop were generally positive, with some recommendations for improvement. Specific positive comments, as related to each of the learning objectives, are provided below.

Responses for “What did you like about this workshop?” included the following:

• Objective 1: Describe the history of Haiti and Haitians within the United States.
  ○ “I enjoyed learning about the history of Haiti which gives a cultural context to the Haitian patients we see.”
  ○ “It was beautifully designed. Very engaging and I learned a lot! Feeling the love for the Haitian community from Haiti to Brooklyn.”
• Objective 2: Compare and contrast health issues and disparities of Haitians residing in Haiti and the United States.
  ○ “I enjoyed the cases; they were a good way to really look at the disparities in practice.”
  ○ “Excellent, clear, concise description of the history of Haiti and the environmental political factors contributing to the health disparities. Cases tied directly back to the content of the slides.”
• Objective 3: Explain how at least two implemented policies have affected Haitians’ and Haitian Americans’ health outcomes.
  ○ “I love the integration of policy, history, and health. It gives me a better understanding of Haitian health.”
• Objective 4: Describe at least two health care access problems faced by Haitians in the United States and/or Haiti.
  ○ “Different/various approaches to understanding the background of Haitian patients.”
  ○ “Workshop was very informative and highlighted every aspect in improving care for Haitian patients.”

In answer to the question “What suggestions do you have to improve this workshop?”, several respondents requested additional time, making comments such as “more time to go over the great information,” and suggested placing key information on busy slides in bold text and providing slides to participants ahead of the workshop for prereview. Most importantly, respondents suggested the addition of real Haitian-identified patients who could share their health conditions and issues in relation to the US and Haitian health care system.

## Discussion

Because the Haitian population in the United States is the largest outside of Haiti, this workshop was designed to educate diverse health care trainees and professionals about the historical, political, and social factors that contribute to Haitian health. Trainees and health professionals gain a structural competency for the Haitian community that will enable them to provide the best care for the Haitian patient. The workshop helps to address the lack of material related to Haitian health care disparities and helps to fill a knowledge gap regarding cultural awareness of Haitian and Haitian American patients. Notably, the workshop is presented applying methods that center on cultural values, history, governmental policies, and a current portrayal of Haitian patients. Accordingly, the learning objectives are for participants to comprehend the history of Haiti and Haitian identity within the United States, compare and contrast health issues and disparities of Haitians residing in Haiti and the United States, explain how policies have impacted the health outcomes of Haitians in Haiti and/or the United States, and describe health care access problems faced by Haitians in Haiti and/or the United States. A comparison of Haitian and Haitian American communities recognizes the migratory patterns that exist within Haitian families and the complexity in ensuring quality care.

Based on the quantitative and qualitative feedback responses, the objectives were overwhelmingly met. In general, the presentation was very well received, and there were statistically significant increases in participant knowledge. Additionally, improvements were made to the workshop based on the suggestions shared in the evaluations.

Ideally, our workshop is designed for a small to midsize audience of fewer than 40 individuals. The suggested audience size has been selected to ensure active participation and group reflection with discussion. We have found and believe that a smaller audience promotes a more energetic and engaging environment for discourse. In addition, facilitators should also consider a lecture hall layout to promote intimate small-group discussions and participant interactions. However, recognizing that many medical school classes are larger in number, the facilitator can promote an interactive environment by situating students in small groups to discuss cases before calling the whole group together to participate in a discussion.

A number of qualities made this workshop effective. First, most participants shared that the case activity was an interactive way to open conversation around the intersection of culture, health, and history. Some thought the hypothetical case scenarios made the content more engaging, while others said applying cultural context to practical examples made the presentation more informative. Second, participants appreciated the video interview that discussed the scales of trauma, which provided additional visuals that helped establish a deeper understanding of cultural familiarity. Additionally, most participants noted that they valued the presentation's organization, delivery, and relevance to a familiar patient population.

There were also opportunities for improvement. For presentation structure, the most common recommendation was to reduce the amount of information on the individual slides. To address this, we restructured slides that were content heavy. Some participants desired more content on the current care of Haitian and Haitian American patients. Other participants desired us to incorporate live interaction with Haitian patients who could exemplify a real-world health care experience. The ability to do this is of course limited and requires an ongoing trusting relationship with the Haitian community. If a facilitator of this presentation does in fact have such a relationship, then we encourage the inclusion of a willing participant to provide the patient perspective. However, we do not believe one is necessary to meet the objectives of the module. Additionally, other participants requested best practices for providers to become more culturally competent for their Haitian patients. While we understand this desire, the aim should not be to master the Haitian culture but rather to achieve a baseline understanding that incorporates the ideals of cultural humility. There is no one lecture that would provide all cultural norms of a given community, nor do we need to master all norms to provide culturally sensitive care.

In summary, this workshop addresses a need for curricula dedicated to the Haitian community, which represents a significant portion of the Black immigrant community. Although the workshop focuses on the Haitian community, the methodology of understanding a community through structural and social determinants of health with the addition of cultural and historical perspectives is one that should be utilized for any community being served. Vulnerable, marginalized, and minoritized communities are not monolithic, and understanding the unique contributions and barriers to care of each is important.

We created an interactive workshop to investigate the intersection of history, culture, policy, and the social determinants of health. Medical students and health care practitioners are influenced by the effects of history and politics, often in ways many might not understand. These effects of history and politics are present in discourse about policy reform, ahistorical maligning of immigrant and Black communities, and the downstream activities of providing care to the most vulnerable communities. Our workshop integrates individual and community aspects with societal factors using vignettes. The discussion and solutions are framed to address all levels from the individual to the institutional to the community. Students and practitioners can draw from their own experiences and interests to address how best to care for the Haitian community using any one individual level or through a multipronged approach. Our students and practitioners are left with a foundational knowledge of not only how to best interact with diverse communities with cultural humility but also how to evaluate different communities and patients in order to understand the internal and external forces that impact health.

During the development of this module, special care was taken to create a multidisciplinary work group. All members of the work group are of Haitian heritage and have worked with or for the Haitian community in the United States and/or Haiti. This allowed us to integrate both experiential and evidence-based perspectives on how best to care for the Haitian patient and community. The work group members’ knowledge and experience relating both to Haitian heritages and to health care students and professionals in the United States allowed for the development of a module that can be facilitated in diverse settings.

Through participant feedback from the postworkshop survey, we were able to identify strong characteristics of the presentation and implement changes to improve the delivery and comprehension of the presented information. Many participants shared their appreciation of the presentation's interactive nature along with the relevant content and illustrations. Additionally, participants appreciated the applicability of the case presentations as examples of current issues experienced by Haitian patients in medical centers. Providing hypothetical experiences encouraged participants to apply the information presented throughout the module to real issues. Given the dynamic nature of health care, facilitators can also consider sharing their own professional experiences in caring for patients who identify as Haitian. Facilitators’ comments should align with the objectives of this module. This will allow participants to understand the relevance and importance of cultural competence in modern medicine and as a caregiver when new issues arise.

There were some limitations present in the design of this module. Though the presentation was well received, it is imperative to note that it provided a single session with self-assessment surveys and multiple-choice questions given immediately before and after the presentation. No assessment of change in behavior or attitude was done. Change in attendees’ competency in Haitian-identified patients’ health care issues may not be sustained and would need to be reevaluated in the future. For example, further follow-up in assessing maintained competency could occur after 6 months. Additionally, it is important to account for the impact of the changing political landscape and its impact on Haitian and Haitian American health care with regard to immigration status, access and availability to care, and government intervention impacting the community. As health care continues to evolve, newly developed perspectives, including stereotypes and bias, may arise that could influence the delivery of care. For that reason, reviewing up-to-date information before implementing the module is crucial to ensuring an accurate present outlook on Haitian and Haitian American health.

Racial and ethnic considerations including health policy, barriers to care, and bias disproportionately affect underserved populations; thus, a diverse, well-informed physician workforce is required. This would offer the knowledge to address components on all levels from the individual to the institutional and community through both practice and policy. This workshop enables participants to introduce these topics and encourages them to proactively become involved in one or more levels of intervention.

## Appendices


Presentation.pptxFacilitator Guide.docxVideo Trauma in the Haitian Context.mp4Case Presentations.docxPre- and Postworkshop Evaluation Form.docx
*All appendices are peer reviewed as integral parts of the Original Publication.*
