# Supplementary material for: An Introduction to Structural Competency for Haitian-Identified Patients: History, Culture, and Access to Care
Source: MedEdPORTAL. 2021 Dec 27;17:11207. doi: 10.15766/mep_2374-8265.11207 (PMC8710435; doi:10.15766/mep_2374-8265.11207)
Supplement: Supplementary file 1 — Presentation.pptxFacilitator Guide.docxVideo Trauma in the Haitian Context.mp4Case Presentations.docxPre- and Postworkshop Evaluation Form.docx [file mep_2374-8265.11207-s001.zip › B. Facilitator Guide.docx]

**An Introduction to Structural Competency for Haitian Identified Patients: History, Culture, and Access to Care**

**Appendix B. Facilitator Guide**

**Overall Goals**

The goals of this module are to help medical students, residents, and other health professionals understand and analyze health disparities, access to care, and how identity influences the health status of the Haitian patients, both in Haiti and the United States. This module may be implemented by any healthcare professional.

**Workshop Learning Objectives**

Describe the history of Haiti and Haitians within the United States;

Understand the health issues and disparities of Haitians residing in Haiti and in the United States;

Explain how, at least, two policies have impacted the health outcomes of Haitians in the United States and/or Haiti;

Describe, at least, two health care access problems faced by Haitians in the United States and/or Haiti.

**Workshop Handouts and Materials**

- Pre- and Post-Evaluation Forms
- Computer set up with audio capability, and connection to projector
- Case Discussion Guides
- Cases
- Video: Trauma Informed Care in the Haitian Context

**Suggested Agenda and Timeline**

- Pre-workshop evaluation: 3 minutes.
- Slides 1- 5: Introduction, Objectives, Goals & Agenda- 5 minutes
- Slides 6-13: Haiti location, Haitian history, and culture- 5 minutes
- Slides 14-20: U.S. Haiti relations- 5 minutes
- Slides 21-29: Health Status of Haitians: Haitian concept of health and health in Haiti- 7 minutes
- Slides 30-36: Health status of Haitians: Haitian health status in the united states- 7 minutes
- Slide 37: Trauma informed care in Haitian context: 4 minutes
- Slide 38: Barriers to care- 2 minutes
- Slides 39: Case discussion exercises introduction, 1 minute; Breakout 5 minutes
- Slides 40: Vanessa Case Discussion- 5 minutes
- Slides 41: Jean Case Discussion- 5 minutes
- Slide 42: Delivering Quality care to Haitian communities- 3 minutes
- Slide 43: Take Home points- 2 minutes
- Question & Answer
- Post-workshop evaluation: 3 minutes

**Slide Instructions**

**Slide 1**:

Add names and background information of facilitators to slide.

The facilitators should introduce themselves to the audience and discuss their roles in their respective institutions.

**Slide 2: Goal**

The overarching goal of this workshop is for the attendants to develop a structural competency as it relates to caring for the Haitian community. This entails moving away from a strictly biomedical model of care, to one that understands the determinants of health for the Haitian community which encompasses its history & culture, policy, and the social determinants of health.

This presentation also aims to dispel myths that propagate stigma and bias towards the Haitian community.

**Slide 3: Learning Objectives- 1 min 30 sec**.

The United States and Haiti have a long history of relations. Migration from Haiti to the United States has significantly increased overtime. Haitians are the second highest immigrant group of black ethnicities in the United States, with the first being from Jamaica(citation).

*For these reasons, it is important for us to understand what impacts the health of Haitian communities, and how we can best care for them.*

*The learning objectives of this module are to:*

- *Describe the history of Haiti and Haitians within the United States;*
- *Understand the health issues and disparities of Haitians residing in Haiti and in the United States;*
- *Explain how, at least, two implemented policies have affected Haitians’ and Haitian/Americans’ health outcomes.*
- *Describe at least, two health care access problems faced by Haitians in the United States and/or Haiti.*

**Slide 4:** **Agenda**

*For the next hour we will:*

- *Introduce Haitian history, culture, and traditions;*
- *Discuss U.S.-Haiti relations, including Haitian migration to the United States;*
- *Discuss health statistics and healthcare of Haitians in Haiti and the United States;*
- *Discuss some cases and reflect on them;*
- *Finish with some recommendations to health care providers on delivering quality care to the Haitian community.*

**Slide 5: Disclaimer**

Before we begin, it is important that we ‘set the stage’ for this presentation. It is important to understand that Haitians are not a monolithic group and has diversity in race, ethnicity, and socioeconomic status.

Haitian history and culture play a role in all, no matter the race or socioeconomic status.

This module will primarily focus on the most marginalized and underserved and was developed through a social justice and equity lens.

**Slide 6:**

Haiti is an island nation located on the western third of the island of Hispaniola

Population as of 2016 was nearly 11million.

**Slide 7: Haitian history and culture introduction slide**

We shall start with Haitian history and culture;

Haiti has a unique history that has shaped its society, families, individuals, and view of the world.

**Slide 8: Land Acknowledgement**

Before we delve in, let us take a moment to acknowledge the original inhabitants of the island of Hispaniola, the Arawak/Taino Indians.

The name ‘Ayiti’ was given by the Arawak/Taino Indians, which means ‘Land of the Mountains.’

**Slide 9: Haitian History: pre-independence**

In 1492 Christopher Columbus arrived to ‘Ayiti’ while looking for the East Indies. The Tainos were used as slaves and they quickly decimated by diseases brought in by the Spaniards and by violence.

Importation of slaved began around 1577.

In **1640**, France sent their Governor to claim the island, and Spain ceded one third of the island to France.

The island of Hispaniola was know as the ‘Pearl of the Antilles;’ Frances most profitable colony.

In **1793,** Toussaint L’Ouverture, a former slave led a slave revolt against France.

Napoleon was unsuccessful in his bid to reclaim Haiti which led to Haiti declaring its independence on **January 1, 1804**.

Notably, as a result of the Haitian revolution, Napoleon signed the Louisiana Purchase treaty which doubled the size of the United States at the time.

**Slide 10: Haitian history: Post independence**

Following its independence, Haiti was forced to pay reparations to France. The modern equivalence is $21 billion. These payments crippled the Haitian economy and negatively impact development.

This led to decades of instability which led to the United States to invade Haiti in **1915**. During this time, the United States played a role in developing economy and the Haitian army.

In **1956**, “Papa Doc” Francois Duvalier took began his dictatorship by organizing a military coup. He declared himself as the Haitian president for life.

In **1971,** Duvalier died and was succeeded by his son Jean-Claude “Baby Doc” Duvalier. However, He fled from Haiti in **1986** following a rebellion.

In **1990,** Jean- Bertrand Aristide became the first democratically elected president of Haiti. One year following his election a coup d’état was led against him. He would eventually be was restored to his position then re-elected to his position in the years following.

Post Aristide, Haiti has been subjugated by financial and political instability with intervention by both the United States and United Nations.

**Slide 11:**

Before we move on from history, we felt that it was important to present the importance of Haiti to United States history because of the negative stigma around Haiti. Some examples are:

- The Louisiana purchase which as we said doubled the size of the United States;
- The city of Chicago was founded by a Haitian immigrant;
- WEB Dubois was the son of a Haitian immigrant;
- The largest unit of soldiers of African descent who fought in the American revolution were from Haiti. There is a statue honoring this in South Carolina.

And Last but not least,

- Toussaint L’Ouveture is featured in the Museum of African American history in the Hall of Founding Fathers, as the ‘Father for Freedom’ for his role in the Haitian revolution, which was the first successful slave rebellion.

**Slide 12: Haitian Culture**

Haitian culture is a blend of Taino, African, and European traditions

The official languages are Haitian Creole and French. It is spoken by majority of Haitians, as opposed to French.

The majority of Haitians identify as Catholic, and a smaller percentage Protestant. Voudou, which is a mixture of traditional African religion and Catholicism has 60 million followers. Because of the negative stereotypes associated with Voudou, many are less likely to report they follow Voudou.

**Slide 13: Haitian Cuisine**

Much like culture, Haitian cuisine is a mixture of Taino, African and European influences

Haitian cuisine is known for its oily meats.

*Fun fact*- Barbecue comes from the Taino cuisine Barbacoa.

- Taino’s brought corn and root vegetables like Yuca;
- The African influence includes rice and Okra;
- French influence includes Bread, cheese, and dessert.

Haitian diet does include many items high in starch and fried food which increases the risk of chronic health issues.

**Slide 14: U.S. Haiti relations**

Our next section will focus on U.S. Haiti relations. United States and Haiti have a long and objectively mixed relationship. They were the first and second free republics of the Western Hemisphere, and as such have a long history of relations.

The determinants of health this will focus on are policy & immigration.

*Fun fact:* The Haitian embassy was the most visit embassy in Washington D.C., hosting many cultural events open to the public. The photo in the PowerPoint is the Haitian embassy in Washington D.C.

**Slide 15: U.S. – Haiti Relations**

The United States and Haiti have had mixed relations since its independence in 1804 which has had political and financial impact on Haiti’s development.

The United States private sector has had a growing role in Haiti. U.S. based companied manufacturing clothing is currently Haiti’s top export.

Foreign aid has had an impact in Haiti- both positive and negative.

One of the biggest successes has been the **U.S. President's Emergency Plan for AIDS relief (PEPFAR)** which originally signed by George W. Bush. This was the largest commitment by any nation to address a single disease. This policy has contributed 90 billion in funding globally. IN Haiti, it has facilitated the fight against HIV/AIDS and has enabled improvement in other aspects of health.

Another program is USAID which is a U.S. State Dept initiative that provides development support to countries.

As discussed, while there have been many initiatives with positive impact, there have been negative as well. For example, U.S. funding from both the government and private flow primarily through not-for-profit organizations (NGO) which weakens the government.

Another example is food aid, which benefits American farmers through subsidies, but has had a negative impact on Haitian farmers.

**Slide 16: Haitian migration to the U.S.**

The U.S. is home to the largest Haitian migrant population.

According to the 2010 U.S. census, Haitians are the second highest reported among foreign born blacks.

The Haitian diaspora, are an important part of the Haitian economy accounting for 25% of the country’s GDP.

**Slide 17: Haitian Migration to the U.S.**

As demonstrated on the table to the right, the Haitian immigrant population has steadily increased over the past decades.

There have been three mass waves of immigration.

The first started in the 1950’s and was the result of a **DICTATORSHIP** of Papa Doc.

These immigrants were more likely to be of higher socioeconomic status and professionals which led to a ‘Brain drain;’

The 2^nd^ was a result of the dictatorship of ‘Baby Doc’ and increased political instability;

The 3^rd^ resulted from political instability and poverty and was more likely to be immigrants of lower socioeconomic status.

**Slide 18: Immigration policy**

As it relates to immigration policy, there have been wide ranging policy from the Post-Mariel policy in **1980** which labeled ‘Cuban-Haitian’ entrants to become possible legal residents, to an interdiction policy which supported the search of vessels. The most recent immigrant policy which impacts patients is the Temporary Protected Status.

The **Temporary protected status** grants work permits and time limited protection from deportation.

***It was granted following the 2010 earthquake***

Currently, 50,000 Haitian are under this program and they are eligible for health insurance.

They also contribute significantly to our economy with TPS holders being employed at high rates and contribute significantly to the U.S. GDP.

**Slide 19: Current Immigration challenges**

The current immigration challenges that the Haitian community faces and can impact their health are:

**1) Public Charge rul**e: this rule allowed the Trump administration to deny green cards to immigrants who could potentially use public benefits. This has become a deterrence to immigrants accessing care.

**2) Temporary protected status:** The Trump administration desired to end the Temporary protected status.

**3) Remain in Mexico policy:** Some Haitians attempt to migrate to the U.S. through Mexico and are affected by this policy

**Slide 20: U.S. policy and Haitian Health**

Now that we have discussed policy and immigration, we will take a moment to summarize it in a manner that connects it to Haitian health

- First, what policy has affected health in Haiti:
  1. PEPFAR: this policy has had tremendous positive impact in Haiti and its ability to combat HIV/AIDS;
  2. Food aid: which has had a NEGATIVE impact because it has led to economic losses for Haitian farmers;
  3. Immigration policy.
- Policy in the United States:
  1. Public charge: as we mentioned has a **NEGATIVE** impact since it acts as a deterrence;
  2. Medicaid policy: Medicaid policy is different depending on the state you live. IN NY, the Medicaid policy is comparatively ‘friendly’ to immigrants as it has less stringent criteria for coverage, which of course is positive.
- Policy that impacts both Haitians in the United States and Haiti would be Immigration policy and Temporary protected status.

**Slide 21: Transition slide: Health status of Haitians**

In this next section we will focus on the Health status of Haitians covering, the Haitian concept of Health, Health in Haiti, Impact of the Earthquake and Health in the United States

**Slide 22: Haitian concept of Health**

The traditional concept of health is based on balance and equilibrium of many factors including:

- Familial relationships
- Spiritual well-being
- Illness is often believed to be a result of wrongdoing
- Many Haitians believe that God is the ultimate decider of health and illness.
  - This may be manifested as ‘passivity’ concerning health decisions
  - Another characteristic that may present itself as ‘passivity’ is the general high regard for Doctors that Haitians have. There is a well-known saying that means “After God is Doctor”

**Slide 23: Clinical correlation**

We wanted to share clinical examples:

A 43-year-old woman presents for follow up visit. Two years ago, she had a high-grade abnormal pap test. She did not present for colposcopy as scheduled. When asked why she has not followed up she states “I believe in God I do not need the test”

As her clinician, how would you interact with this patient?

What methodologies can you use?

**Slide 24: Health in Haiti**

Haiti, is unfortunately the most impoverished nation in the Western Hemisphere, with 65% of the population living below the poverty line.

As it relates to the health system, 47% of the Haitian people lack access to basic health services.

Poverty, the social determinants of health and structural inequity contribute to poor health outcomes

The Health system is fractured, and has financial, human resource and material challenges.

Haiti also has the highest per-capita rate of NGO’s in the world. 75% of healthcare is provided by NGO’s. This has both positive and negative effects on health in Haiti. Many NGO’s provide great support and care in Haiti, but much of international funding flows through NGO’s which weakens the government and government health institutions.

As a result, Haiti has much of the worst health outcomes in the Western Hemisphere including Malnutrition, Maternal and Infant mortality, and life expectancy.

**Slide 25: 2010 Earthquake**

We cannot talk about Health in Haiti without discussing the earthquake

In 2010, Haiti experienced the most powerful earthquake to hit the country in 200 years.

It had a significant impact on both lives lost and the healthcare system with 30 of the 49 health facilities in the area being damaged and more then 220,00 lives lost.

Later that year the Cholera outbreak began which killed more than 9,000 people.

The earthquake also had a significant negative impact on mental health. Following the earthquake, the prevalence of Post-traumatic stress disorder was 24.6% and Major Depressive disorder was high among survivors.

The impact of the earthquake also affected those in the diaspora with increased rates of PTSD, anxiety, and depression.

**Slide 26:** **Infectious Diseases**

Vaccine preventable diseases remain prevalent secondary inadequate coverage

Major infectious diseases that are still prevalent include: HIV/AIDS, TB, Cholera, food and waterborne diseases, vector borne diseases such as Dengue fever and malaria and water contact diseases

**Slide 27: Causes of Mortality in Haiti**

Over the past ten plus years we have seen an epidemiologic shift in causes of mortality in Haiti.

The table on the left demonstrates the top ten causes of mortality in 2007. We see that in 2007, HIV/AIDS was the number one cause of HIV/AIDS and infectious disorders are on the list.

The table on the right demonstrates the top ten causes in 2017 with the corresponding percent change. The table shows that infectious diseases like HIV/AIDS decreased as a cause for overall mortality with Chronic diseases becoming more prevalent as causes of mortality.

**Slide 28: Risk factors that drive the most death and disability**

If we look at the risk factors that cause death and disability, we see a similar trend.

In 2007 we see that risk factors that lead to infectious diseases are higher on the list, compared to 2017 where we see risk factors like high fasting glucose which contribute to chronic diseases increasing in prevalence.

**Slide 29: Trend in health indicators**

Although the statistics in Haiti are not good, if we take a step back on a global view there have been overall improvement over the past couple of decades.

In this table we see Infant and under five mortality. We see improvement over the past couple of decades. The decrease did slow after 2006, and without direct evidence, we suspect this is likely related to the 2010 earthquake.

**Slide 30: Haitian health status in the United States**

Now we will transition to health status in the United States.

Starting on a global level, in the United States, poor health outcomes disproportionately affect communities of color leading to Health disparities. Health disparities are preventable differences in health outcomes between populations.

Evidence has shown that racism, Inequity, and the Social determinants of health contribute to these health disparities.

So, this picture of already existing disparities in health outcomes for communities of color are further complicated for recent immigrants that can be related to being of a lower socioeconomic status or barriers related to being foreign born.

**Slide 31: Haitian immigrant community demographics**

The median income is $47,200.

57% of the Haitian immigrant community are naturalized citizens.

This community plays a significant role in the civilian labor force. Interestingly, Haitian immigrant women are more likely to be in the labor force then the overall female immigrant population.

**Slide 32: Haitian Health Status in the United states**

In the United States, the Haitian community is often demographically grouped with African American ethnicities for epidemiological research purposes. Haitians are therefore poorly sampled by large surveys because of relatively small numbers. Additionally, Haitians are concentrated in a relatively few metropolitan areas. As mentioned before, the largest communities are in Miami, Florida, New York City, and Boston.

Most research work has been on illnesses, especially HIV, cervical cancer, TB, and the health impacts of the earthquake.

Now, we are going to provide a brief overview of health conditions that affect the Haitian community, and in which published literature exist. Additionally, wherever applicable, we will discuss the health condition from a global view as is relates to health disparities and then focus on the Haitian community. Lastly, if exists, we will present documented barriers as it relates to that health condition.

**Slide 33: HIV**

First, we will start with HIV/AIDS

The HIV/AIDS epidemic is one of the significant contributors of stigma in the Haitian community. At the height of the epidemic, the CDC unscientifically labeled as a risk group for HIV.

In general, U.S. immigrants of Caribbean origin are overrepresented in HIV/AIDS prevalence statistics. Haitians are also more likely than African Americans to be hospitalized for HIV related complications and are more likely to delay seeking care.

Heterosexual contact is the most prevalent mode of transmission.

The most likely contributing factors towards the prevalence are risk at country of origin, social circumstances in the U.S, gender inequities, limited healthcare access and social isolation.

**Slide 34: Preventative care**

When we examine preventative care among the Haitian immigrant community in Miami, Florida it was found that HTN and DM were among most prevalent health conditions,

Factors that were associated with service utilization included: insurance coverage, established care, educational attainment, household income, and duration of residency. Overall, citizenship status was the strongest independent predictor of service utilization.

**Slide 35: Cervical Cancer**

Next, we move on to Cervical cancer. Cervical cancer is the 4^th^ most common cancer in women

In the United States, although overall cervical cancer rates have declined, African American women have higher mortality rates.

If we look at Haitian women, this group experiences an increased risk of developing and dying from Cervical cancer, as compared to U.S. born Black women. Additionally, compared to U.S. born Blacks, it has been shown that Haitian women are less likely to receive Cervical cancer screening.

One group who have studied barriers to screening among Haitian immigrants (that lead to disparities in screening) developed a theoretical model, categorizing barriers as either: Structural, Psychosocial, or Sociocultural.

Structural factors are those that limit a women’s ability to seek out screening and include lack of insurance, language, and financial considerations, as examples;

Psychosocial factors include fear and modesty;

Sociocultural factors: beliefs that define illnesses as symptomatic.

**Slide 36: Severe Maternal Morbidity**

- If we look at maternal outcomes, women of color suffer disproportionately form pregnancy related deaths and poor outcomes
- Black women have three times the rate of Severe Maternal Morbidity (SMM) then white women. SMM is a life-threatening complication during delivery
- In NYC, women born in Haiti have the highest SMM rate
- Chronic stress, which can be from social stressors, racism or discrimination is believed to play a contributory role in adverse outcomes in communities of color
- Additionally, women with obesity and chronic medical conditions are increased risk of SMM

**Slide 37: Global Trauma Research**

When the creators of this module discussed what would be important to highlight, universally it was felt that highlighting Trauma within the Haitian community and its effects were important.

The Haitian community is one that has experienced multiple levels of trauma.

Dr. Florence Saint Jean, the Executive Director of Global Trauma Research is going to talk to us through this vide on the impact of Trauma in the Haitian community.

**Slide 38:**

As we try to conceptualize and organize what we have learned we can try to understand the barriers of care for the Haitian community we can organize into three levels:

First Race: These are barriers that persons of color have historically and still currently experience. They include:

- Structural racism (the unfair distribution of wealth, power and resources)
- social determinants of health (where we live, work, play),
- Racism and Bias.

All these factors have been shown to negatively impact communities of color

Next, we add the Immigrant aspect. Barriers to care for immigrant communities include:

- Unfamiliarity and difficulty navigating the system
- Lack of insurance
- Fear of immigration status
- Distrust in care
- Language barriers

Now, based on what we have learned from this module we can add another aspect as it relates to the Haitian community. Barriers specific to this community include:

- **First, Language, Language, Language.** Although language will be a barrier for many immigrant communities, in the Haitian context there are unique barriers. Haitian Kreyol began as an oral language. There is a lack of linguistic equivalence for many health-related terms.
- **Stigma/bias**- the Haitian community has unfortunately been recipient of stigma.and bias which has negatively affected the community
- **Lack of familiarity with preventative services**- which may reflect lack of such services in Haiti
- The sociocultural aspect of “better not to know”
- Preference for natural remedies

**Slide 39: Case Presentation introduction**

Now we will proceed to discuss some cases that bring into focus some of the situations that we have been mentioning.

*Note: the facilitator will decide if the cases will be discussed in the large group setting (slides are available as part of the PPP). The facilitator may use one or more cases.*

*As an alternative, the group may be divided into three small groups and each will discuss one case (see Case Discussion Guide). Groups will present answers to guided questions at the end of the discussion time.*

Allow 20 minutes for discussion in groups.

For each case, please reflect on the following:

- The health problems of the patient.
- The risk factors contributing to the health problems.
- Any problems with access to health care.
- Different ways to improve the health care the patient is receiving.

**Slide 40-41: Case Presentations**

Read the slides

**Slide 42: Delivering Quality Care to Haitian Communities**

Let us reflect on the following questions for each of our cases:

***First Vanessa***

What health problems does Vanessa present? (provide a minute for the audience to answer)

Not a problem, but first she is pregnant

- Obesity
- Diabetes
- History of Cesarean Delivery

What were the risk factors contributing to these problems or difficulty in managing? (provide a minute for the audience to answer)

- Cultural? what are food norms
- Socioeconomic factors including work schedule, caretaker, supporting family

Can you identify any problems Vanessa may have with access to health care? (provide a minute for the audience to answer)

- Prenatal visits are not convenient, work schedule is a barrier
- Financials
- Language/cultural- both during pregnancy and following when her baby is in the NICU

What do you think could be done to improve the health care Vanessa is receiving? (provide a minute for the audience to answer)

- Patient centered prenatal care- that would be easier for Vanessa to access care if hours are convenient for working community
- Culturally appropriate nutrition counseling
- Increase access to support services
- Address language/cultural barriers

***Now let's move on to Jean***

Let us Reflect on the following questions:

What health problems does Jean present? (provide a minute for the audience to answer)

- Diabetes
- Hypertension
- Congestive Heart Failure
- Probable Post Traumatic Stress Disorder

What were the risk factors contributing to these problems? (provide a minute for the audience to answer)

- Poorly controlled PTSD due to likely access and different cultural norms
- Trauma as a result of Natural disaster.

Can you identify any problems Jean may have with access to health care? (provide a minute for the audience to answer)

- Immigrant status
- Language & Cultural- No term for mental health in Haitian Kreyol. Need for culturally appropriate, trauma informed medical and mental health care.
- Financial- Cost of medication and equipment needed to properly control chronic disease is expensive
- Difficulty navigating a new health care system.
- Fatalism: influencing attitude on seeking proactive care

Religion and spirituality are an essential part of Haitian life and culture. Religion among Haitians is multifaceted with a combination of Haitian spirituality, African religious practices and Catholicism. Most Haitians identify as Roman Catholic and Protestant. The concept of health among Haitians is strongly connected to spirituality.

What do you think could be done to improve the health care Jean is receiving? (provide a minute for the audience to answer)

- Family focused intervention- this can be especially helpful as it relates to PTSD and trauma
- Trauma informed medical and mental health care
- Social support and navigation services

**Now that we have examined each case on the Individual levels, it is important that we now examine and provide recommendations on a community level.**

On a community level, how do you think an individual, healthcare center or institution can help to deliver quality care to Haitian communities? (provide a minute for the audience to answer)

- Cultural humility- incorporates a lifelong commitment to self-evaluation and self-critique, addressing the power imbalances in patient-physician dynamic and to developing mutually beneficial clinical and advocacy partnerships with communities.
- Outreach through Haitian community networks (ex. Faith based groups)
- Advocate- when we say advocate, we do not just mean just at the legislator level. We believe that there is a role for advocacy at all levels- individual, health center, health institution, community, local and national. On an individual or practice/health center level, this means advocating on behalf of your patient or community you serve for the best practice and/or policies in your practice.
  - Note for facilitator: you can ask the audience for examples. Example can include, having culturally appropriate information material

**Slide 43: Take Home Points**

As a final point, lest summarize some recommendations for improving the healthcare of Haitians and the Haitian community.

The Haitian community is diverse with a rich history and culture that informs the view of the world and themselves.

Stigma has and continues to play a role in bias towards the Haitian community.

Haitian health disparities exist within black racial disparities.

We cannot overstate the importance of **LANGUAGE**. There does not exist direct translation for many healthcare terms. Additionally, **CULTURAL** **TRANSLATION** is very important.

Having presence of Haitian staff if servicing a Haitian community is essential.

Trauma informed care is important as trauma can impact health and ability to manage health conditions.

Interpersonal connection is always important, and you can increase comfort by saying “Sak Passe” which is a popular phrase that means “what's up.”

**Slide 44-45:**

We would like to thank all the contributors who worked on this module, and all that supported this effort.
