# Supplementary material for: An Introduction to Structural Competency for Haitian-Identified Patients: History, Culture, and Access to Care
Source: MedEdPORTAL. 2021 Dec 27;17:11207. doi: 10.15766/mep_2374-8265.11207 (PMC8710435; doi:10.15766/mep_2374-8265.11207)
Supplement: Supplementary file 1 — Presentation.pptxFacilitator Guide.docxVideo Trauma in the Haitian Context.mp4Case Presentations.docxPre- and Postworkshop Evaluation Form.docx [file mep_2374-8265.11207-s001.zip › D. Case Presentations.docx]

**An Introduction to Structural Competency for Haitian Identified Patients: History, Culture, and Access to Care**

**Appendix D. Case Presentations**

**Case Presentation: Jean**

Jean is a 55-year-old cis-male born in Haiti. He moved to the United States as a temporary resident following the devastating earthquake in 2010. Jean was stuck under the rubbles for a day until he was finally rescued.  Since leaving his home he has had difficulty adjusting and navigating his new life in New York and has become depressed.  Jean's family, some of whom are in the United States try's to be supportive but are not always able to understand his mood and actions.

Jean suffers from uncontrolled DM (A1C at 11%) and HTN.  His medical conditions have worsened and progressed to diabetic neuropathy and CHF.  Jean has been especially having difficulty with his diabetes medication and has frequent hypoglycemic events.

When you ask Jean about his difficulty controlling his medical illnesses, he confides that he feels a sense of shame being in New York. He also reports worsening backache which is affecting his mobility. When asked about the experience in Haiti related to earthquake, Jean simply responds “that is just a part of life in Haiti”

**Case Presentation: Vanessa**

Vanessa is a 27-year-old cis-female from Haiti who is currently 8 months pregnant with obesity and diabetes. Vanessa has one previous cesarean delivery which was an emergency. She has a three-year-old child at home.

“This time around, I want to do everything right,” She says during one of her prenatal care visits. “I am checking my sugar and eating the right things. I hope everything goes well,” she explains using a *phone interpreter*.

Vanessa tries hard to manage her diabetes and prenatal care but has difficulty keeping her appointments, especially since she requires several appointments weekly. She works as a nursing assistant in a nursing home. Her husband works as a security officer in an office building in Manhattan. “Things are tough. We work very hard to pay the bills.” She has help from family to help her with her child, but she is the main caretaker.

Vanessa’s parents still live in Haiti and are financially reliant on her.

Vanessa underwent an emergency cesarean delivery for fetal distress. Her baby is in the NICU and she visits daily.

Vanessa wants to know how she can help her baby while in the NICU, but has difficulty understanding the status and plan for her baby.

**Questions**

**Vanessa:**

What health problems does Vanessa present?

What were the risk factors contributing to these problems or difficulty in managing?

Can you identify any problems Vanessa may have with access to health care?

What do you think could be done to improve the health care Vanessa is receiving?

**Jean:**

What health problems does Jean present?

What were the risk factors contributing to these problems?

Can you identify any problems Jean may have with access to health care?

What do you think could be done to improve the health care Jean is receiving?

**Jean and Vanessa:**

On a community level, how do you think an individual, healthcare center or institution can help to deliver quality care to Haitian communities?
