# Supplementary material for: An Introduction to Structural Competency for Haitian-Identified Patients: History, Culture, and Access to Care
Source: MedEdPORTAL. 2021 Dec 27;17:11207. doi: 10.15766/mep_2374-8265.11207 (PMC8710435; doi:10.15766/mep_2374-8265.11207)
Supplement: Supplementary file 1 — Presentation.pptxFacilitator Guide.docxVideo Trauma in the Haitian Context.mp4Case Presentations.docxPre- and Postworkshop Evaluation Form.docx [file mep_2374-8265.11207-s001.zip › E. Pre- and Postworkshop Evaluation Form.docx]

**An Introduction to Structural Competency for Haitian Identified Patients: History, Culture, and Access to Care**

**Pre-Test**

**Part I. Demographic Information**

**Select ONE circle that describes your current professional role:**

- Medical Student
- Medical Resident or Fellow
- Academic Faculty (MD, DO, NP, PA, Other)
- Clinician in Non-Academic Practice (MD, DO, NP, PA, Other)
- Other _________________

**Please mark all that apply:**

- Female
- Male
- Transgender or Gender Nonconforming
- Other

**Please mark all that apply:**

- Latino/Hispanic
- Black/African American
- White
- Asian
- Native American/Alaskan or Pacific Islander
- Other ________________

**Part II. Self-Assessment**

| **Please rate how much CONFIDENCE do you have in your ability to…** | **No**  **Confidence**  **0** | **1** | **2** | **3** | **Complete**  **Confidence**  **4** |
| --- | --- | --- | --- | --- | --- |
| Obj 1: Describe the history of Haiti and Haitian Identity within the United States | 0 | 1 | 2 | 3 | 4 |
| Obj 2: List the health issues and disparities of Haitians residing in Haiti and in the United States | 0 | 1 | 2 | 3 | 4 |
| Obj 3: Explain how two implemented policies have affected Haitians’ and Haitian/Americans’ health outcomes. | 0 | 1 | 2 | 3 | 4 |
| Obj 4: Describe two health care access problems faced by Haitians in Haiti and/or the United States | 0 | 1 | 2 | 3 | 4 |

**Part III. Objective Assessment**

**Please Choose the Best Answer:**

1. The **FIRST** wave of immigrants from Haiti resulted from:
   a. A hurricane
   b. A dictator
   c. Financial insecurity
   d. An earthquake
2. This U.S. **POLICY** has had a significant positive health impact in Haiti:
   a. USAID
   b. Food aid
   c. PEPFAR
   d. Trade policy
3. How would you describe the change in causes of mortality in Haiti over the past 20 years?
   a. Increase in cholera
   b. Increase in HIV
   c. Decrease in TB
   d. Decrease in HIV

**An Introduction to Structural Competency for Haitian Identified Patients: History, Culture, and Access to Care**

**Post-Test**

**Part I. Self-Assessment**

| **Please rate how much CONFIDENCE do you have in your ability to…** | **No**  **Confidence**  **0** | **1** | **2** | **3** | **Complete**  **Confidence**  **4** |
| --- | --- | --- | --- | --- | --- |
| Obj 1: Describe the history of Haiti and Haitian Identity within the United States | 0 | 1 | 2 | 3 | 4 |
| Obj 2: List the health issues and disparities of Haitians residing in Haiti and in the United States | 0 | 1 | 2 | 3 | 4 |
| Obj 3: Explain how two implemented policies have affected Haitians’ and Haitian/Americans’ health outcomes. | 0 | 1 | 2 | 3 | 4 |
| Obj 4: Describe two health care access problems faced by Haitians in Haiti and/or the United States | 0 | 1 | 2 | 3 | 4 |

**Part II. Objective Assessment**

**Please Choose the Best Answer:**

1. The **FIRST** wave of immigrants from Haiti resulted from:
   a. A hurricane
   b. A dictator
   c. Financial insecurity
   d. An earthquake
2. This U.S. **POLICY** has had a significant positive health impact in Haiti:
   a. USAID
   b. Food aid
   c. PEPFAR
   d. Trade policy
3. How would you describe the change in causes of mortality in Haiti over the past 20 years?
   a. Increase in cholera
   b. Increase in HIV
   c. Decrease in TB
   d. Decrease in HIV

**Part III. Comments**

**Please answer the following questions:**

1. What did you like about this workshop?
2. What suggestions do you have to improve this workshop?
